# *De novo* mutations in *PLXND1* and *REV3L* cause Möbius syndrome

**DOI:** 10.1038/ncomms8199

**Published:** 2015-06-12

**Authors:** Laura Tomas-Roca, Anastasia Tsaalbi-Shtylik, Jacob G. Jansen, Manvendra K. Singh, Jonathan A. Epstein, Umut Altunoglu, Harriette Verzijl, Laura Soria, Ellen van Beusekom, Tony Roscioli, Zafar Iqbal, Christian Gilissen, Alexander Hoischen, Arjan P. M. de Brouwer, Corrie Erasmus, Dirk Schubert, Han Brunner, Antonio Pérez Aytés, Faustino Marin, Pilar Aroca, Hülya Kayserili, Arturo Carta, Niels de Wind, George W. Padberg, Hans van Bokhoven

**Affiliations:** 1 Department of Human Genetics, Radboud University Medical Center, Donders Institute for Brain, Cognition and Behaviour, PO Box 9101, Nijmegen 6500 HB, The Netherlands; 2 Department of Human Anatomy and Psychobiology, School of Medicine, University of Murcia, 30100 Espinardo (Murcia), Spain; 3 Department of Human Genetics, Leiden University Medical Center, P.O. Box 9600, 2300 RC Leiden, The Netherlands; 4 Department of Cell and Developmental Biology, Cardiovascular Institute, Perelman School of Medicine at the University of Pennsylvania, 9-105 SCTR, 3400 Civic Center Boulevard, Philadelphia, Pennsylvania 19104, USA; 5 Signature Research Program in Cardiovascular and Metabolic Disorders, Duke-NUS Graduate Medical School Singapore, National Heart Center Singapore, 8 College Road, Singapore 169857, Singapore; 6 Medical Genetics Department, Istanbul Medical Faculty, Istanbul University, Millet Caddesi, Capa, Fatih 34093, Turkey; 7 Department of Neurology, Radboud University Medical Center, Donders Institute for Brain, Cognition and Behaviour, PO Box 9101, Nijmegen 6500 HB, The Netherlands; 8 The Kinghorn Centre for Clinical Genomics, Garvan Institute of Medical Research, Sydney, New South Wales 2010, Australia; 9 Department of Human Genetics, Radboud University Medical Center, Radboud Institute for Molecular Life Sciences (RIMLS), PO Box 9101, Nijmegen 6500 HB, The Netherlands; 10 Department of Cognitive Neuroscience, Radboud University Medical Center, Donders Institute for Brain, Cognition and Behaviour, PO Box 9101, Nijmegen 6500 HB, The Netherlands; 11 Department of Clinical Genetics, Maastricht University Medical Center, PO Box 5800, Maastricht 6200AZ, The Netherlands; 12 Dysmorphology and Reproductive Genetics Unit, Moebius Syndrome Foundation of Spain, University Hospital LA FE, Valencia 46540, Spain; 13 Ophthalmology Unit, Department of Biomedical, Biotechnological and Translational Sciences (S.Bi.Bi.T.), University of Parma, via Gramsci 14, 43126, Parma, Italy

## Abstract

Möbius syndrome (MBS) is a neurological disorder that is characterized by paralysis of the facial nerves and variable other congenital anomalies. The aetiology of this syndrome has been enigmatic since the initial descriptions by von Graefe in 1880 and by Möbius in 1888, and it has been debated for decades whether MBS has a genetic or a non-genetic aetiology. Here, we report *de novo* mutations affecting two genes, *PLXND1* and *REV3L* in MBS patients. PLXND1 and REV3L represent totally unrelated pathways involved in hindbrain development: neural migration and DNA translesion synthesis, essential for the replication of endogenously damaged DNA, respectively. Interestingly, analysis of *Plxnd1* and *Rev3l* mutant mice shows that disruption of these separate pathways converge at the facial branchiomotor nucleus, affecting either motoneuron migration or proliferation. The finding that *PLXND1* and *REV3L* mutations are responsible for a proportion of MBS patients suggests that *de novo* mutations in other genes might account for other MBS patients.

## Abstract (web summary)

lt has been debated for decades if there is a genetic aetiology underlying Möbius syndrome, a neurological disorder characterized by facial paralysis. Here Tomas-Roca *et al*. use exome sequencing and identify *de novo* mutations in *PLXND1* and *REV3L*, representing converging pathways in hindbrain development.

## Graphical abstract

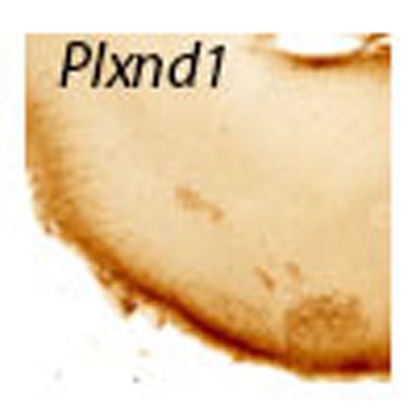

Möbius syndrome (MBS) (MIM 157900) is a rare congenital cranial dysinnervation disorder characterized by non-progressive facial palsy and impairment of ocular abduction, due to paralysis or weakness of the facial (n7) and abducens (n6) nerves, and frequently other cranial nerves^1^,^2^,^3^,^4^,^5^,^6^. Both intrauterine environmental factors and genetic causes have been proposed for the aetiology and pathogenesis of MBS. A disruption of blood vessel migration during development, which can be secondary to misoprostol^7^ or cocaine^8^ exposure during pregnancy, has been hypothesized to lead to hindbrain hypoxia, resulting in cranial nerve dysfunction^9^. A limited number of familial cases with an atypical manifestation of MBS have been reported^10^,^11^,^12^,^13^. In addition, a founder mutation in *HOXB1* has been associated with an autosomal recessive form of an MBS-like syndrome, with limited phenotypic overlap with typical MBS^14^. However, the vast majority of patients has a sporadic occurrence with a very low recurrence risk for siblings. This is consistent with either the involvement of environmental factors or with dominant *de novo* mutations as the underlying mechanism^15^.

The broad spectrum of neuropathological and neuroradiological findings suggests that MBS is due to a developmental defect of the entire rhombencephalon^16^,^17^, rather than an isolated cranial nerve disruption. This is in contrast to hereditary congenital facial palsy (HCFP), which is characterized by the isolated dysfunction of the facial nerve. Two HCFP loci have been identified: HCFP1 (MIM 601471) at chromosome 3q21-q22 (ref. 18) and HCFP2 (MIM 604185) at chromosome 10q21.3-q22.1 (ref. 19). Extensive studies have been conducted to explain the HCFP phenotype and to pave the way for genetic studies in MBS, but analysis of genes in the HCFP1 and HCFP2 loci, including the prime candidate gene *PLXND1* at chromosome 3q21-q22 (ref. 20), has so far not identified any causative mutations in HCFP and in MBS patients^21^.

In this study we undertake exome sequencing of two case–parent trios and six isolated patients with classical MBS to investigate the possibility of an underlying genetic cause. We identify *de novo* mutations in five different genes. For two of these genes, *PLXND1* and *REV3L*, additional *de novo* mutations in other MBS patients are identified. The causality of *de novo PLXND1* and *REV3L* mutations for the neuropathological features of MBS is further supported by analysis of the respective knockout mice. Strikingly, for both heterozygous mutants we observe hypoplasia of the facial branchiomotor nucleus, which is consistent with the facial nerve weakness in MBS patients. Taken together, the present data establish *de novo* mutations as a cause for MBS, providing a rationale for exome sequencing in patient–parent trios to identify *de novo* mutations in other genes underlying MBS.

## Results

### Identification of *de novo* mutations by exome sequencing

**Figure 1:**
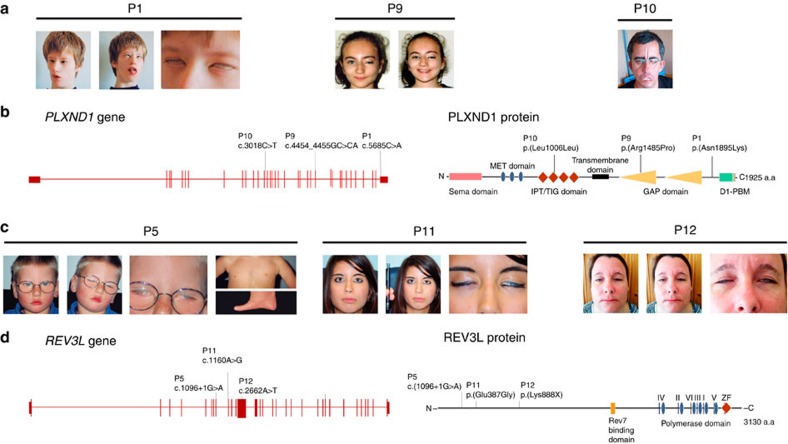
Clinical features of Möbius syndrome patients and *de novo* mutations in *PLXND1* and *REV3L*. (**a**) Clinical features of the patients carrying the mutations in *PLXND1* (P1, P9 and P10). Patient P1 shows an impairment of ocular abduction in a relaxed facial position; she is not able to smile and she cannot close her eyes completely. Patient P9 in a relaxed facial position presents left upper facial paralysis. Patient P10 showing bilateral facial paralysis, inability to close his mouth and lagophthalmos. (**b**) Genomic structure of the *PLXND1* gene and the PLXND1 protein structure and the position of *de novo* mutations. The different protein domains are depicted. GAP, GTPase-activating protein; IPT/TIG, immunoglobulin-like fold, plexins, transcription factors/transcription factor immunoglobin; D1-PBM, PDZ-binding motif; MET, mesenchymal–epithelial transition. (**c**) Clinical features of the patients carrying the mutations in *REV3L* (P5, P11 and P12). Patient P5 in a relaxed position, showing the characteristic inability of MBS patients to smile in the second photograph. Both eyes show incomplete closure. There is agenesis of the left pectoralis muscle (Poland variant) and absence of digits from the left hand. Patient P11 has both eyes fixed in straight position and a complete deficiency of both abduction and adduction. There is a complete inability to follow objects laterally. Furthermore, a bilateral facial nerve palsy is present producing a lagophthalmos on eye closure in both eyes, and oral rim asymmetry with an inability to smile. Patient P12 in a relaxed facial position. The ability to smile of P12 has been improved following plastic surgery at age of 13 years. Inappropriate closure of both eyes is still present. (**d**) Schematic structure of human *REV3L* gene (left) and REV3L protein (right). MBS-associated mutations detected in three patients (P5, P11 and P12) are indicated. The coloured bands represent the known domains of the protein. Rev7-binding domain, site of binding of the heterodimeric Rev3l partner Rev7; ZF, zinc finger.

We excluded all variants inherited from either parent in the two trios under the hypothesis of *de novo* mutations as the underlying cause of MBS (Supplementary Table 1). Two *de novo* variants were detected in patient 1 (P1) (Fig. 1a; reported previously^17^), one in the *SPO11* gene (c.712T>C; p.Cys238Arg; NM_012444) and the other in the *PLXND1* gene (c.5685C>A; p.Asn1895Lys; NM_015103) (Fig. 1a,b; Supplementary Fig. 1; Supplementary Tables 2 and 3). *SPO11* is involved in meiotic recombination, and targeted disruption of this gene in mice results in fertility problems^22^, but no features related to MBS. The occurrence of a *de novo* mutation in *PLXND1* was more consistent with a role for this gene in MBS, given that *PLXND1* was previously considered a strong candidate for HCFP1 and MBS^20^,^21^. Trio-exome sequencing in patient 2 did not reveal any *de novo* variant. A different analytical approach was taken for the six patients with isolated MBS and this was to Sanger sequence all candidate variants identified in the exome studies (Supplementary Tables 1 and 4). Sanger sequencing confirmed *de novo* variants, identified by exome sequencing, in *IFT172*, *CCDC160* and *REV3L*, in three unrelated patients. The *de novo* missense mutation in the *IFT172* gene (NM_015662) identified in patient 3 (Fig. 1; Supplementary Tables 2 and 3) is unlikely to be causative for the MBS phenotype, because biallelic mutations are associated with the unrelated Jeune and Mainzer-Saldino Syndromes in humans^23^. The *de novo* variant in the X-chromosomal *CCDC160* gene of patient 4 (P4) (a male) is a c.501delA frameshift mutation in exon 2 (NM_001101357) (Supplementary Fig. 1; Supplementary Tables 2 and 3). Patient 5 (P5; Fig. 1c) carries a *de novo* variant in *REV3L*, c.1096+1G>A, affecting the canonical donor splice site in exon 10 (NM_002912) (Fig. 1d; Supplementary Fig. 1, Supplementary Tables 2 and 3). Aberrant splicing products were identified by quantitative reverse transcriptase–PCR analysis of *REV3L* messenger RNA obtained from Epstein-Barr virus-transformed lymphoblastoid cell lines (EBV-LCLs) of patient P5. Sequencing of these products revealed aberrant *REV3L* transcripts lacking either exon 9 or exons 9 and 10, in addition to decreased levels of normal *REV3L* transcript (Supplementary Fig. 2).

### Additional *de novo* mutations in the *PLXND1* and *REV3L*

Next, we screened *CCDC160*, *PLXND1* and *REV3L* by Sanger sequencing in a cohort of 103 MBS patients, including some HCFP patients who were not previously tested for *PLXND1* (ref. 21). The screening identified four additional *de novo* mutations in unrelated patients, two affecting *PLXND1* and two affecting *REV3L* (Fig. 1; Supplementary Fig. 1; Supplementary Tables 2 and 3). The *PLXND1* mutations were identified in patients P9 (c.4454_4455GC>CA; p.Arg1485Pro) and P10 (c.3018C>T; p.Leu1006Leu). The mutation in P9 affects a conserved arginine residue in the GTPase-activating protein domain of the protein. The silent mutation in P10 is at a leucine residue in the IPT/TIG domain (immunoglobulin-like fold, plexins, transcription factors/transcription factor immunoglobin) that is characteristic for plexin proteins^24^. The two additional *de novo* mutations in *REV3L* were identified in patients P11 and P12. A *de novo* missense mutation c.1160A>G was identified in patient P11, predicting an amino-acid substitution p.Glu387Gly. In addition, patient P12 carried a nonsense mutation (c.2662A>T; p.Lys888*), which is predicted to result in a loss-of-function allele.

The absence of *PLXND1*, *REV3L* and *CCDC160* truncating variants in NHLBI Exome Sequencing Project (ESP; http://evs.gs.washington.edu/EVS/) and a low residual variant intolerance score for these genes of −1.30 (4.88th percentile), −2.1 (1.55th percentile) and 0.014 (54.98th percentile), respectively, are consistent with *PLXND1*, *REV3L* and *CCDC160* being intolerant to loss-of-function mutations^25^. The amino acids that are affected by the missense variants in *REV3L* and *PLXND1* are highly conserved throughout evolution (p.Glu387Gly (P11), p.Asn1895Lys (P1), p.Arg1485Pro (P9) and p.Leu1006Leu (P10)) (Supplementary Fig. 3).

The probability of identifying multiple *de novo* mutations in the same gene in a cohort of 103 individuals was calculated using the Poisson test and is 0.0001217 for *REV3L* (*P* value=2.4 × 10^−4^) and 9.34e−05 for *PLXND1* (*P* value=1.8 × 10^−4^). The presence of a mutation in the other allele in these patients was excluded by sequencing the coding exons of *PLXND1* and *REV3L*. Screening of *CCDC160* gene in our cohort of male MBS patients did not reveal additional *de novo* mutations.

### *PLXND1* deficiency affects neural fibres' structures

**Figure 2:**
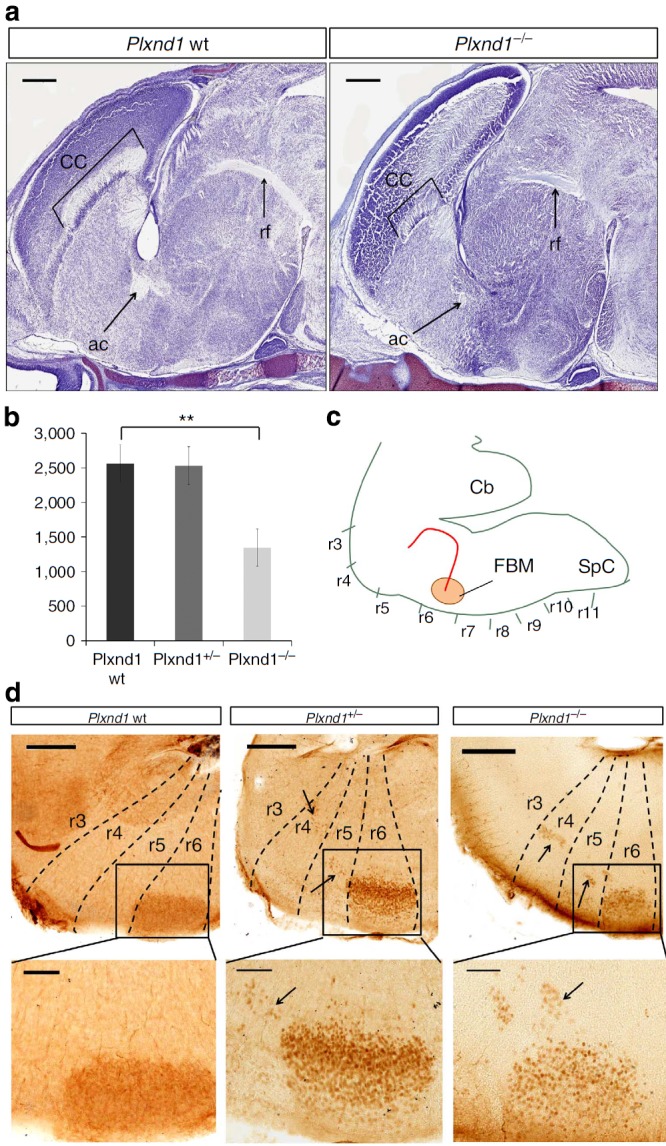
*Plxnd1* mouse brain characterization. (**a**) Paraffin sagittal sections of embryonic mouse brains processed with Nissl staining at E16.5. These two pictures are medial sections of a view of the corpus callosum (CC), the anterior commisure (ac) and the fasciculus retroflexus (rf). These three structures appear hypoplastic in *Plxnd1*^−/−^ mice. Scale bars, 500 μm. (**b**) Graphic representation of the number of motoneurons in facial motor nucleus in wt, heterozygous and homozygous *Plxnd1* knockout (mean±s.d.). Unpaired *t*-test was used to calculate the *P* value=0.0049 (*N*=5). (**c**) Schematic representation of the facial nerve migratory process along the hindbrain. The motoneuron migratory pathway is indicated with a red arrow. The rhombomeres (r3–r11) boundaries are marked with a line. Cb, cerebellum; FBM, facial motor nucleus; SpC, spinal cord. The upper part in (**d**) shows the immunohistochemical staining of facial FBM motoneurons with anti-Islet-1 antibody (dark brown). The rhombomere units (r3–r7) are marked with a dashed line. Black arrows point the motoneurons migration across the rhombomeres in the heterozygous and the homozygous *Plxnd1* knockout mouse. The area of the facial motor nucleus is marked by the square. Scale bar, 300 μm. A detailed view of the facial motor nucleus and motoneurons migration is shown in the lower part of **d**. Motoneurons appear outside the facial nucleus along the migratory path of the facial nerve in both the heterozygous and the homozygous genotypes. Scale bar, 100 μm. All reported *P* values tested were calculated by the unpaired *t*-test using Graphpad software.

*PLXND1* encodes a protein of 521 amino acids and is a member of the plexin family of proteins. Plexins bind to semaphorins (Semas), a large family of extracellular, secreted and membrane proteins^26^,^27^. *PLXND1* is expressed in the vascular system^20^ and in specific structures of the central nervous system, such as the cranial and spinal ganglia, the cortical plate, the external granular layer of the cerebellum, the striatum and in dendritic cells^28^,^29^,^30^. Sema3E/PlexinD1 signalling is involved in the development of two descending forebrain tracts, the corticofugal and the striatonigral tracts^31^. To determine the effect of deficiency of *PLXND1* in central nervous system development, we compared morphologic structures of the embryonic brain of wild-type (wt), heterozygous and homozygous *PlexinD1* knockout mice at E16.5 (ref. 32). Histological comparison of the three genotypes revealed consistent hypoplasia of the corpus callosum, anterior commissure and fasciculus retroflexus in *Plxnd1*^−/−^ mice (Fig. 2a). These three brain structures are bundles of neural fibres that connect brain areas. Both the corpus callosum and the anterior commissure allow interhemispheric communication, whereas the fasciculus retroflexus links the forebrain with the midbrain.

### *Plxnd1* mutant mice show a defect of neuronal migration

The facial nerve phenotype in *Plxnd1* heterozygous and homozygous mice was investigated. Analogous to observations in brain autopsies of an MBS patient^3^, we observed a significant decrease in the number of motoneurons in the facial branchiomotor nucleus in homozygous mice as compared with wt mice (Unpaired *t*-test *P* value=0.0049; Fig. 2b). The development of the cranial nerve undergoes a complex migration within the rodent hindbrain^33^ (Fig. 2c). Between E10.5 and E14.5, neurons in rhombomere 4 (r4) start migrating tangentially along the ventral midline, reaching r5 and r6. Then they migrate dorsally until the alar/basal plate boundary and begin to migrate radially to their final destination in the pial surface of r6 (refs 33, 34, 35). To trace the nature of the decreased neuronal density of the FBM, we carried out an immunohistochemical analysis using the motoneuron-specific molecular marker Islet-1 in brains from E16.5 embryos (Fig. 2d). In line with this migration pattern, all FBM motoneurons reached r6 at E16.5 in wt mice (Fig. 2d). In sharp contrast, in all of the *Plxnd1*^+/−^ and *Plxnd1*^−/−^ mutant mice, a subpopulation of motoneurons was still located in r4 and r5 at E16.5. Collectively, these results support a causative role of a defect in motoneuron migration caused by a heterozygous *Plxnd1* mutation akin to the *de novo PLXND1* mutations in MBS patients P1, P9 and P10.

### *Rev3l* heterozygous mice show hypoplasia of neural structures

**Figure 3:**
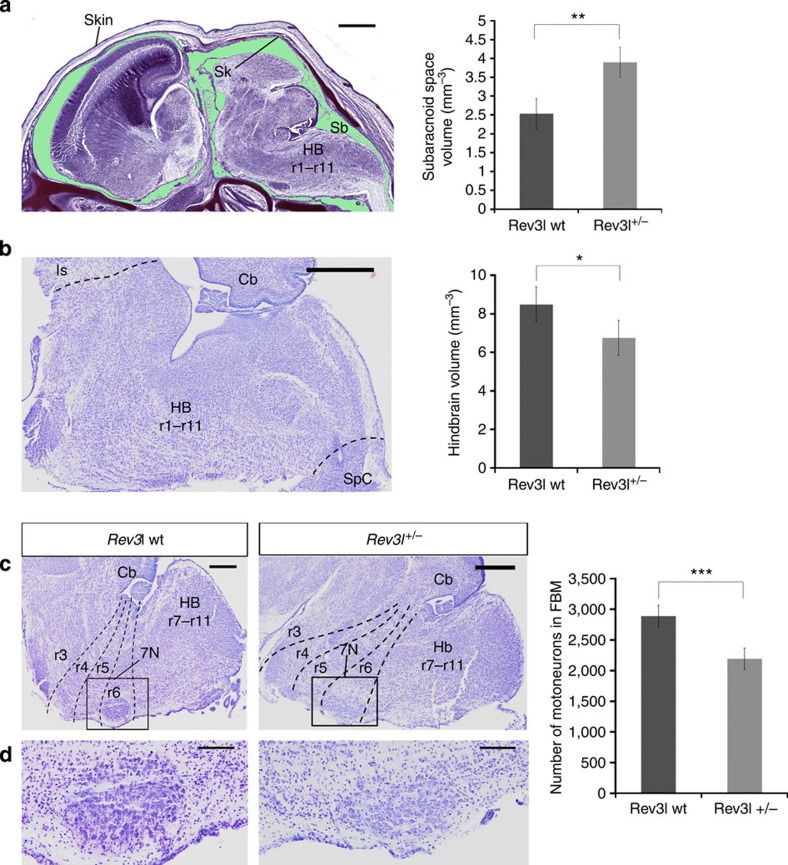
Morphological analysis of the brains of *Rev3l* embryonic and newborn mice. (**a**) Example of a sagittal section of the embryonic mouse head at E16.5 stage used for subarachnoid volume rendering, processed with Nissl staining. The subarachnoid space is shown in green. Scale bar, 1 mm. Right: subarachnoid volume rendering measures of both genotypes. In all *Rev3l* heterozygous embryos the subarachnoid space is significantly increased as compared with wt embryos (Unpaired *t*-test *P* value=0.0047, *N*=5). Scale bar, 500 μm (**b**) Example of one of the hindbrain sagittal sections at P0 used for hindbrain volume rendering. We measured the volume of five wt and five heterozygous mice. The hindbrain boundaries are indicated by dashed lines. Scale bar, 500 μm. Right: *Rev3l* heterozygous mice show a significant decrease of hindbrain volume (Unpaired *t*-test *P* value=0.015, *N*=5). (**c**) Lateral hindbrain sections at the P0 stage of a view of the facial motor nucleus, inside the square. Scale bar, 300 μm. (**d**) Higher magnification of the facial motor nucleus showing paucity of *Rev3l* heterozygous motoneurons. Scale bar, 100 μm. Right: quantification of motoneurons in the facial motor nucleus, in each side of the hindbrain, in five wt and five heterozygous P0 mice (mean±s.d.). The difference is statistically highly significant (Unpaired *t*-test *P* value<0.0001, *N*=10). We do not show both genotypes because there are no visible differences between them. 5N, trigeminal nucleus; 7N, facial nucleus; Cb, cerebellum; HB, hindbrain; Is, isthmus; r1–r11, rhombomeres r1 to r11; Sb, subarachnoid space; Sk, skull; SpC, spinal cord. A *P* value smaller than 0.05 was considered to be statistically significant (*). *P* values smaller than 0.01 (**) or 0.001 (***) were considered highly significant.

The human REV3L protein acts as the catalytic subunit of DNA polymerase ζ, a key protein in replication of endogenously or exogenously damaged DNA^36^,^37^. Consistent with the phenotypes of the *Rev3l* heterozygous mice, *in situ* hybridization data show that *Rev3l* is highly expressed in the developing embryonic brain around mid-gestation (Supplementary Fig. 4). Biallelic inactivation of *Rev3l* in mice leads to mid-term embryonic lethality. *Rev3l*-deficient mouse embryos display pleiotropic morphological abnormalities, but are invariably growth-retarded and display massive apoptosis, notably of the brain^36^. Analysis of the subarachnoid space at E16.5 in *Rev3l*^*+/*−^ mice revealed a highly significant increase in subarachnoid volume (*t*-test, *P* value=0.0047; Fig. 3a). In addition, *Rev3l* heterozygous mice at P0 showed significantly reduced hindbrain volumes as compared with wt (*t*-test, *P* value=0.015; Fig. 3b). We then analysed the facial motor nucleus at P0 (Fig. 3c,d). In contrast to the *Plxnd1* mutant mice, there was no evidence for a motoneuron migration defect in the *Rev3l* heterozygous embryos. Nevertheless, there was a strong decrease in the number of motoneurons in *Rev3l* heterozygous embryos (*t*-test, *P* value<0.0001; Fig. 3d). These data provide strong support for hypoplasia of the motor nucleus consequent to a heterozygous *Rev3l* mutation, similar as the *REV3L* mutation in MBS patients P5, P11 and P12.

### *Rev3l* mutant MEFs displayed increase of DNA damage

**Figure 4:**
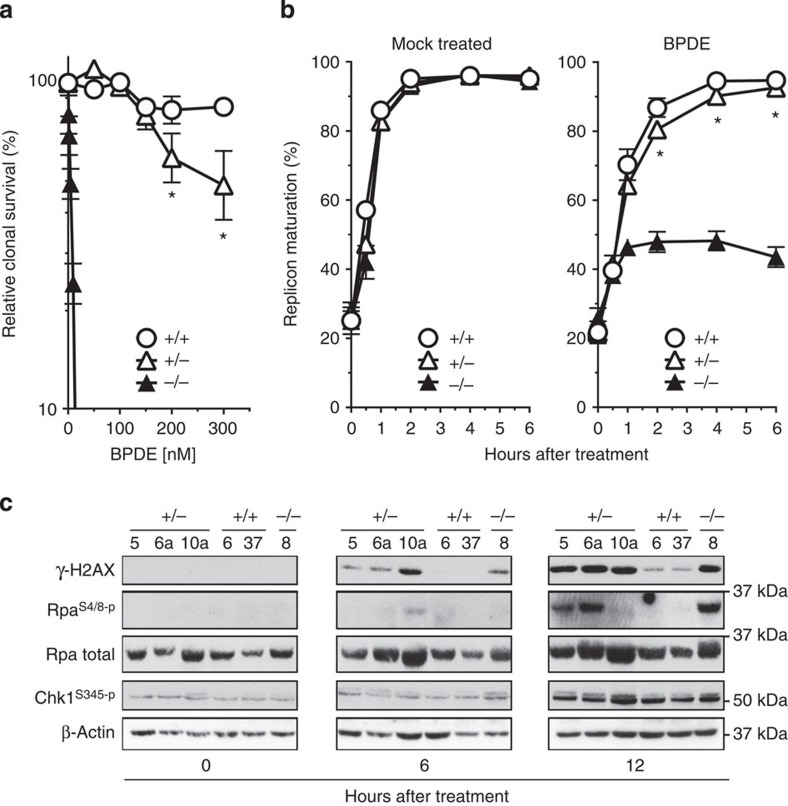
Survival, replication of bulky DNA lesions and DNA damage signaling in Rev3l-mutated MEF lines. (**a**) Survival of wt (+/+), *Rev3l* heterozygous (+/−) and *Rev3l*-deficient (−/−) MEF lines after mock exposure, or after exposure to Benzo(a)pyrene diolepoxyde (BPDE). *Rev3l* heterozygous MEFs are slightly sensitive to BPDE indicating haploinsufficiency. Experiments were performed in triplicate. Error bars, s.e.m. The asterisks indicate a significant difference between the wt and the heterozygous lines (unpaired *t*-test, *P*<0.05). (**b**) Representation of replication fork progression after exposure to BPDE, or mock exposure. *Rev3l* heterozygous MEFs display normal fork progression at undamaged DNA (mock treated, left panel), but a marginal defect in replication of bulky lesion-containing DNA (BPDE, right panel). Error bars, s.e.m. (**c**) Immunoblots to detect the phosphorylation of DNA damage-signalling markers upon treatment with BPDE. γ-H2AX: phosphorylated histone H2AX. Rpa^S4/8-P^: 32 kDa subunit of single-stranded DNA-binding protein Rpa, phosphorylated at Ser 4 and/or Ser 8. Total levels of Rpa are somewhat variable consequent to slight differences in proliferation rate between the lines. Chk1^S354-P^ Checkpoint kinase-1, phosphorylated at Ser 354. β-actin: loading control. *Rev3l*-heterozygous MEFs display DNA damage signalling that is almost as strong as *Rev3l*-deficient MEFs.

The essential role of Rev3l in replication of damaged DNA has been extensively studied *in vitro* and in *Rev3l*-deficient cells and mice^36^,^37^,^38^,^39^. We wondered whether the neuronal defects in MBS patients and *Rev3l* heterozygous mice reflect partial replication defects at DNA carrying endogenous DNA lesions. To this aim, we analysed responses of immortalized *Rev3l* mutant mouse embryonic fibroblasts (MEFs) to Benzo[α]pyrene diolexpoxide (BPDE), an agent that induces DNA damage that mimics bulky endogenous nucleotide lesions. *Rev3l* heterozygous MEFs displayed a slight, but significant, decrease in cell survival (Fig. 4a). To substantiate the effect of haploinsufficiency, we measured replication of BPDE-damaged DNA in these cells and found that *Rev3l* heterozygous cells displayed a slight but significant defect, suggesting the presence of replication stress (Fig. 4b, right panel). Since replication stress induces DNA damage-signalling cascades that initiate cellular senescence or apoptosis, we measured phosphorylation of the signalling proteins Rpa, Chk1 and H2AX (Fig. 4c)^40^. Remarkably, the *Rev3l* heterozygous cells displayed a level of DNA damage signalling that was almost as high as in *Rev3l*-deficient cells. These data suggest that, in *Rev3l* heterozygous mice, marginally under-replicated endogenous DNA lesions induce strong DNA responses, contributing to the observed neuronal phenotypes.

## Discussion

We have identified *de novo* mutations in *PLXND1* and *REV3L* in six unrelated sporadic MBS patients. The neuropathological alterations and other clinical findings in MBS patients correlate to those in the *Plxnd1* and *Rev3l* mutant models. Thus, hypoplasia of the facial motor nucleus that was observed in both mouse models has also been documented in MBS patients^3^,^16^. In addition, several developmental anomalies that were previously reported in *Plxnd1* knockout mice are mirrored in patients carrying a *PLXND1* mutation, as well as other MBS patients. For example, craniofacial bone abnormalities observed in *Plxnd1* knockout mice^41^,^42^ were also seen in patients P1 and P9, and which are commonly seen in other MBS patients^17^ (Supplementary Table 5). In addition, the vertebral defects in *Plxnd1* mutant mice are reminiscent of the scoliosis in patient P1, and the haemorrhages seen in *Plxnd1* mutant mouse resemble the facial haemangiomas seen in patient P1 (Supplementary Table 5). A possible causative *de novo* mutation was also identified in *CCDC160.* No *PLXND1*-coding mutations were identified in HCFP1 families, although their genetic defect maps to the *PLXND1* locus^20^, suggesting the presence of regulatory mutations affecting the spatiotemporal expression of *PLXND1* during embryonic brain development.

The clinical features of patients with heterozygous *PLXND1* and *REV3L* mutations are highly variable with no obvious genotype–phenotype correlations. While the variable phenotype of patients P1, P5 and P9–P11 are consistent with the clinical criteria for MBS^6^,^43^, patient P12, who was identified as having a heterozygous nonsense mutation in *REV3L*, only had bilateral facial paralysis and no weakness of the abducens nerves, suggestive of the restricted HCFP phenotype (Fig. 1; Supplementary Table 5). Thus, our data show that MBS and HCFP can be allelic conditions. In contrast to P12, the phenotype of patient P5, who was identified as having a heterozygous loss-of-function splice-site mutation in *REV3L*, presented with a wide range of features in addition to the cranial nerve palsies, including absence of digits on the left hand, Poland anomaly, hearing loss and cardiac defect. This variability is highly reminiscent of the variable phenotype of completely *Rev3l*-deficient embryos. *Rev3l* deficiency may result in a stochastic ablation of cell lineages during embryonic development^36^. On the basis of these and our current data, it is conceivable that the spectrum of MBS symptoms reflects the stochastic loss of neuronal precursors, caused by replicative stress at endogenous DNA lesions and consequent DNA damage responses, during embryonic development. Likewise, the variable clinical presentation that is seen for *PLXND1* mutations might be due to similar stochastic disruptions of motoneuron migration to the FBM nucleus.

Exome sequencing has detected causative *de novo* mutations in two (or three including the *CCDC160* mutation) in the initial cohort of eight MBS patients. This result is of general interest, as several studies have suggested previously that vascular disruptions during the first trimester of pregnancy rather than genetic mechanisms are the leading cause of MBS^5^,^6^,^7^,^44^. While vascular disruptions caused by various teratogens can explain the MBS phenotype in some patients, our current data indicate that exome sequencing in parent–patient trios is a powerful approach for identifying causative mutations in MBS. It is to be expected that whole-genome sequencing will deliver an even higher yield when focusing on *de novo* mutations in coding and noncoding regions^45^. That only three *de novo* mutations were identified in *PLXND1* and three in *REV3L* in our cohort suggests a low frequency of mutations in these genes as a cause of MBS, implying genetic heterogeneity and intrauterine insults as causative events in other MBS patients. We expect that the implementation of whole-exome sequencing and whole-genome sequencing of parent–patient trios in a diagnostic setting will further dissect the aetiology of MBS.

## Methods

### Patients

We identified eight isolated Möbius patients in this study. They were the only affected individuals in their families. These patients (patients 1–8) were selected from a cohort of patients with the pathognomonic signs of MBS^6^,^43^: facial and abducens palsy^17^. Clinical details of these patients and other patients carrying *de novo* mutations (P9, P10, P11 and P12) are given in the Supplementary Table 5 and in Fig. 1a. Informed consent was obtained from all the subjects involved in this study, and all procedures were in accordance with the ethics of the World Medical Association Declaration of Helsinki. Ethical approval for the reported studies was obtained from the Medical Ethical Committee Arnhem-Nijmegen, from the University of Parma, the University of Istanbul and University Hospital LA FE, Valencia.

### Whole-exome sequencing

Exome sequencing was performed on DNA from peripheral blood, using the SureSelect Human All Exon v2 50 Mb Kit (Agilent, Santa Clara, CA) followed by multiplexed analysis on a SOLiD 4 System sequencing slide (Life Technologies, Carlsbad, CA) as described previously^46^. Colour space reads were mapped to the hg19 reference genome with the SOLiD BioScope version 1.3 software, which utilizes an iterative mapping approach. Sequence analyses from all the patients are summarized in Supplementary Table 4. Statistical single-nucleotide variants were subsequently called by the Di-Bayes algorithm with high call stringency. Called variants were combined and annotated with a custom analysis pipeline. The total number of variants identified in each patient are summarized in Supplementary Table 1. Of these variants, some were located in the exonic sequences or located in canonical splice sites, defined as the dinucleotides up- and downstream of exonic sequence (Supplementary Table 1).

Under the hypothesis of *de novo* mutations as the underlying cause of MBS syndrome, we excluded all variants inherited from either parent in the two trios (Supplementary Table 1). From the remaining variants in these two patients, we excluded all synonymous variants, as well as variants present in (single-nucleotide polymorphism database) dbSNP (v138) or in our in-house-sequenced exome database (5,031 exomes), and variants in noncoding regions with the exception of canonical splice-site sequences. Manual inspection of mapped reads excluded additional, likely false positive, variants. After Sanger sequencing, only two variants were validated *de novo* mutations in one of the trios.

The approach for the six isolated cases was slightly different. For these, we applied a filtering scheme excluding variants observed in dbSNP (v138) and in our in-house database (5,031 exomes (Supplementary Table 1). We further analysed all truncating variants, missense variants with a PhyloP score >3 or those affecting genes with a possible involvement in MBS, as suggested by the gene ontology term or mouse mutant phenotype. Candidate variants were validated by Sanger sequencing, and parental DNAs were tested for *de novo* occurrence of the respective variant (Supplementary Tables 2 and 3).

The reference gene sequences referred to in this study are available in the RefSeq database under the following accession code: *PLXND1*, NM_015103; *REV3L*, NM_002912; *CCDC160*, NM_001101357; *SPO11*, NM_012444 and *IFT172*, NM_015662.

### Mutation analysis

Primer sequences were designed using Primer3 software (http://frodo.wi.mit.edu/) encompassing the candidate variants. To exclude additional changes in either *REV3L*, *PLXND1* or *CCDC160*, we designed primers for the amplification of all exons and intron–exon boundaries of *REV3L* (Genbank ID NM_002912), *PLXND1* (Genbank ID NM_015103) and *CCDC160* (Genbank ID NM_NM_001101357). Sequences of primers are shown in Supplementary Table 6. PCR was performed on 50 ng of genomic DNA with Taq DNA polymerase (Invitrogen, Carlsbad, CA). A nucleofast 96 PCR plate (Clontech Lab, Mountain View, CA) was used to purify the PCR amplicons, according to the manufacturer's protocol. Sequence analysis was performed with the ABI PRISM Big Dye Terminator Cycle Sequencing V3.1 ready Reaction Kit and the ABI PRISM 3730 DNA Analyzer (Applera Corp, Foster City, CA). PCR products were sequenced by Sanger sequencing and analysed using Vector NTI software (Life Technologies).

### Cell culture

EBV-LCL cells from patient P5 and five controls were cultured to a density of 0.5 × 10^6^ cells per ml. After incubation for 4 h at 37 °C, cells were harvested by centrifugation at 200*g* for 5 min at room temperature, washed once with PBS, pelleted by centrifugation at 200*g* for 5 min at room temperature and snap frozen in liquid nitrogen. MEFs were obtained from E13.5-day-old embryos of intercrossed *Rev3*^*+/*−^, *p53*^*+/*−^ mice. The embryos were finely minced, trypsinized and the resulting MEFs were cultured in DMEM supplemented with 10% fetal calf serum and antibiotics until spontaneous immortalization^47^.

### First-strand synthesis

EBV-LCL cells were harvested by centrifugation at 6,000*g* at room temperature. RNA isolation was performed using the NucleoSpin RNA kit (Qiagen, Hilden, Germany) according to the manufacturer's protocols. RNA was reverse transcribed using the iScript cDNA synthesis kit (Bio-Rad Laboratories, Hercules, CA, USA) according to the manufacturer's protocol. Complementary DNA (cDNA) was purified with NucleoSpin extract II (Qiagen) according to the manufacturer's protocol.

### Quantitative PCR (QPCR) analysis

SYBR green-based real-time QPCR expression analysis was performed on an Applied Biosystem Fast 7,500 System machine. Primer sequences around exon 10 were designed using Primer3 software (http://frodo.wi.mit.edu/) (Supplementary Table 6). Primers encompassed at least one boundary between two exons. *GUSB* was used as a reference gene^48^. QPCR quantifications were performed in duplicate on the equivalent of 5 μg of total RNA from the first-strand synthesis and included a water control. Experimental threshold cycles (Ct) values were within the range of cDNA dilutions used to validate the primers. The melt curves of all PCR products showed a single PCR product. All water controls were negative. Differences in expression of the *REV3L* alleles were calculated by the comparative Ct or 2^ΔΔCt^ method^49^. The *P* value was derived from the standard score (*Z*-value) as compared with the normal distribution of the five controls. We used an *α*-level of 0.05, because only one gene was assessed.

### Reverse transcriptase–PCR

cDNA from patient 1 EBV-LCLs and one control were used to amplify *REV3L*. The first pair of primers was designed surrounding the canonical splice-site mutation (c.1096+1G>A) in exon 10, and a control pair of primers was designed within exon 14 (see Supplementary Table 6).

### Mouse breeding

*Plxnd1* heterozygous mice were maintained on a C57BL/6 background and mated to generate *Plxnd1* knockout and heterozygous embryos (male and female) for analysis. *Plxnd1* null and wt mouse embryos (E16.5) were harvested from timed matings. Noon was designated E0.5. Embryos were genotyped as described^32^. All *Plxnd1* mouse work was performed according to the University of Pennsylvania animal care guidelines.

C57BL/6 wt, *Rev3l* heterozygous and *Rev3l*-deficient mice and embryos were described before^36^. C57Bl/6 *Rev3l* heterozygous mice were mated to generate male and female embryos and newborn mice for analysis. *Rev3l* heterozygous and wt mouse embryos (E16.5) were derived from timed matings. Noon was designated E0.5. Embryos were genotyped as described^36^. Mice were treated using Federation of Laboratory Animal Science Associations (FELASA) standards and mouse experiments were performed under permit DEC 11108 from the animal experiment committee of the Leiden University Medical Center.

### Mouse tissue preparation

Seven *Plxnd1* wt, seven *Plxnd1* heterozygous and seven *Plxnd1* homozygous embryos at E16.5 (stage corroborated according to Theiler^50^) were killed and brains were dissected in PBS at room temperature and fixed in 4% paraformaldehyde at 4 °C overnight. The next day, brains were washed in PBS, dehydrated in ethanol and stored at −20 °C. Brains were processed for paraffin and agarose embedding and sectioned into 16- and 90-μm slices using a microtome and vibratome, respectively.

We analysed two different stages to compare phenotypic aberrations. Five wt and five *Rev3l* heterozygous embryos at E16.5 (stage corroborated according to Theiler^50^) and five wt and five *Rev3l* heterozygous P0 newborn pups were killed and fixed by immersion in phosphate-buffered 4% paraformaldehyde (0.1 M; pH 7.4) at 4 °C for 24 h. Fixed brains and heads were embedded in paraffin and they were sectioned into 16-μm slices using a microtome.

### Tissue staining

Seven *Plxnd1* wt, seven *Plxnd1* heterozygous and seven *Plxnd1* homozygous embryos at E16.5, five *Rev3l* wt and five *Rev3l* heterozygous embryos at E16.5 (stage corroborated according to Theiler^50^) and five *Rev3l* wt and five *Rev3l* heterozygous P0 newborn pups were embedded in paraffin, sectioned and were stained with Nissl stain according to routine protocols^51^.

### Immunohistochemistry

Two *Plxnd1* wt, two *Plxnd1* heterozygous and two *Plxnd1* homozygous embryos were embedded in agarose and processed for immune histochemistry with the Islet-1 antibody (catalogue number 39.4D5) obtained from the Developmental Studies Hybridoma Bank (University of Iowa, Iowa City). Sections were washed in PBS and then treated with 0.1% hydrogen peroxide in PBS for 1 h in the dark to inactivate endogenous peroxidase activity. After several rinses in PBT (PBS with 0.2% Triton X-100), sections were blocked with 0.5% goat serum, 0.2% BSA and 0.2% Triton X-100 (Sigma, St Louis, MO, USA) in PBS for 4 h and then incubated overnight at 4 °C with monoclonal antibody anti-Islet-1 (1:50 in blocking solution). Subsequently, sections were incubated with biotinylated goat anti-mouse secondary antibody (1:300, 2 h incubation; BA-9200, Vector Laboratories, Burlingame, CA, USA), and then with streptavidin/horseradish peroxidase complex (1:200, 2 h incubation; PK-4000, Vectastain-ABC kit; Vector Laboratories). All antibodies were diluted in the same blocking solution as the primary antibody. The histochemical detection of the peroxidase activity was carried out using 0.03% diaminobenzidine and 0.005% H_2_O_2_. After immunoreaction, the sections were mounted, dehydrated and then coverslipped with Eukitt (Fluka, Buchs, Switzerland).

### Quantification of motoneurons

We counted the total number of motoneurons presents in the facial motor nucleus in paraffin sagittal sections processed with Nissl staining. We used five *Plxnd1* wt, five *Plxnd1* heterozygous and five *Plxnd1* homozygous mice at E16.5 and five *Rev3l* wt and five *Rev3l* heterozygous P0 newborns, and we counted separately the number of motoneurons from the right and left hindbrain sides (around 12 sections each side) (20–25 slices total per animal). For the counting we used the open-source ImageJ Cell Counter software^52^. Differences in the number of motoneurons between the heterozygous, homozygous and the wt mice were calculated by a comparative *t*-test, using Graphpad Software (http://graphpad.com/quickcalcs/ttest1.cfm).

### Brain structures volume rendering

We performed volume rendering of image stacks on serial light microscopic sections of 16 μm using the open-source ImageJ TrakEM2 (ref. 53). For the volume rendering of the subarachnoid space around 100 sagittal slices of each of the five *Rev3l* wt and five *Rev3l* heterozygous mice at E16.5 were used. This embryonic stage was selected because the calcification of the cranial bones is not completed yet allowing the sectioning. For hindbrain volumes comparison, around 80 slices of each of the five *Rev3l* wt and five *Rev3l* heterozygous brains at P0 were used. After alignment of all images from each brain respectively, we highlighted the areas of interest in each section to measure their volumes. The boundaries of the hindbrain were delineated according to the Allen Developing Mouse Brain Atlas from rhombomere r1 until r11, including the cerebellum.

### Survival assay

*Rev3l* survival assays were conducted with *Rev3l*^+/+^, *Rev3l*^+/−^ and *Rev3l*^−/−^ MEFs. In each well of a six-well plate, 0.5 × 10^6^ exponentially growing cells were seeded in MEF medium (DMEM supplemented with 10% fetal calf serum, glutamax and antibiotics) and cultured overnight. Then, the cells were exposed to 0–300 nM BPDE (Biochemical Institute for Environmental Carcinogens, Grosshansdorf, Germany) that was dissolved in anhydrous tetrahydrofuran (THF, Sigma-Aldrich) for 60 min in serum-free medium at 37 °C. The cells were trypsinized and seeded in MEF medium at clonal densities in 9-cm dishes. After culturing for 12–14 days at 37 °C, cell clones were fixed, stained with methylene blue and the number of cell clones was counted (*n*=3). The number of clones after mock treatment was set at 100%. The data were obtained with two and three independent *Rev3*^+/+^ and *Rev3*^+/−^ MEF lines, respectively, and one *Rev3*^−/−^ MEF line.

### Replication progression assay

Progression of replicons using the Alkaline DNA unwinding assay was determined following seeding of 5 × 10^4^ exponentially growing cells per well in a 24-well plate and culturing overnight in MEF medium at 37 °C. The cells were mock treated with the solvent THF or exposed to 500 nM BPDE in serum-free medium for 15 min at 37 °C. The medium was removed, cells were washed once with 150 mM NaCl and pulse labelled with [^3^H]thymidine (2 μCi ml^−1^; 76 Ci mmol^−1^) in MEF medium for 15 min at 37 °C. MEF medium with radioactive thymidine was removed and the cells were cultured in MEF medium supplemented with 10 μM cold thymidine. At different times after pulse labelling, the local unwinding of DNA ends of elongating replication forks, the separation of single-stranded DNA and double-stranded DNA (dsDNA) using hydroxylapatite columns and the determination of radioactivity in the fractions containing single-stranded DNA and dsDNA was performed^47^ (*n*=3). The percent of maturation of replicons was calculated by the following equation: (radioactivity in dsDNA/total radioactivity) × 100%. The data were obtained with single *Rev3*^+/+^ and *Rev3*^−/−^ MEF lines and two *Rev3*^+/−^ MEF lines.

### Western blotting

In each well of a six-well plate, 2.5 × 10^5^ exponentially growing cells were seeded in MEF medium and cultured overnight. Then, the cells were mock treated with the solvent THF or exposed to 500 nmol BPDE for 15 min in serum-free medium at 37 °C. After rinsing the cells with PBS, cells were cultured in MEF medium. At different times after treatment, up to 12 h, cells were lysed using Laemmli buffer. The proteins were separated by SDS–polyacrylamide gel electrophoresis and blotted onto nitrocellulose membranes (Hybond-C extra; Amersham Biosciences). The membranes were incubated overnight at 4 °C with antibodies specific for γH2AX (S129) (mouse monoclonal; Millipore; cat. no. 05-0636; dilution 1:1,000), phosphorylated Chk1 (at serine 345; rabbit polyclonal; Cell Signaling; cat. no. 2348; dilution 1:1,000), phosphorylated Rpa32 (at serines 4/8; rabbit polyclonal; Bethyl; cat. no. A300-245A; dilution 1:1,000), total Rpa (rat polyclonal; Cell Signaling; cat. no. 2208; dilution 1:1,000) and β-actin (mouse monoclonal; Oncogene; cat. no. CP01; dilution 1:20,000). After incubation with appropriate secondary antibodies conjugated to peroxidase (Bio-Rad), proteins were visualized using enhanced chemoluminescence detection. Uncropped scans with indicated size markers are shown in Supplementary Fig. 5.

### Statistical analysis

The probability of finding two mutations in a cohort of 103 in each gene was calculated by the Poisson test. We took into account the presence of two alleles and estimated gene mutation rates based on recent exome sequencing data^54^. Moreover, a conservative Bonferroni correction was done for multiple testing.

Statistical significance of differences in motoneuron counts between the wt mice and the mutant mice were determined by the unpaired *t*-test. The significance of differences of the hindbrain volume and subarachnoid space between the *Rev3l* heterozygous and the wt mice was calculated by the comparative *t*-test. Significance in sensitivities of MEF lines to BPDE and in replication fork progression after exposure to BPDE was determined using the unpaired *t*-test, *σ*=0.90. A *P* value smaller than 0.05 was considered to be statistically significant (*), and *P* values smaller than 0.01 (**) or 0.001 (***) were considered highly significant.

## Additional information

**How to cite this article:** Tomas-Roca, L. *et al*. *De novo* mutations in *PLXND1* and *REV3L* cause Möbius syndrome. *Nat. Commun.* 6:7199 doi: 10.1038/ncomms8199 (2015).

The novel exome sequencing data generated in this study has been deposited at the European Genome-phenome Archive (http://www.ebi.ac.uk/ega/) under accession numbers EGAS00001001250.

## Supplementary Material

Supplementary Information Supplementary Figures 1-5, Supplementary Tables 1-6 and Supplementary References
