# Supplementary material for: De novo mutations in PLXND1 and REV3L cause Möbius syndrome
Source: Nat Commun. 2015 Jun 12;6:7199. doi: 10.1038/ncomms8199 (PMC4648025; doi:10.1038/ncomms8199)
Supplement: Supplementary Information — Supplementary Figures 1-5, Supplementary Tables 1-6 and Supplementary References [file ncomms8199-s1.pdf]

Supplementary figures

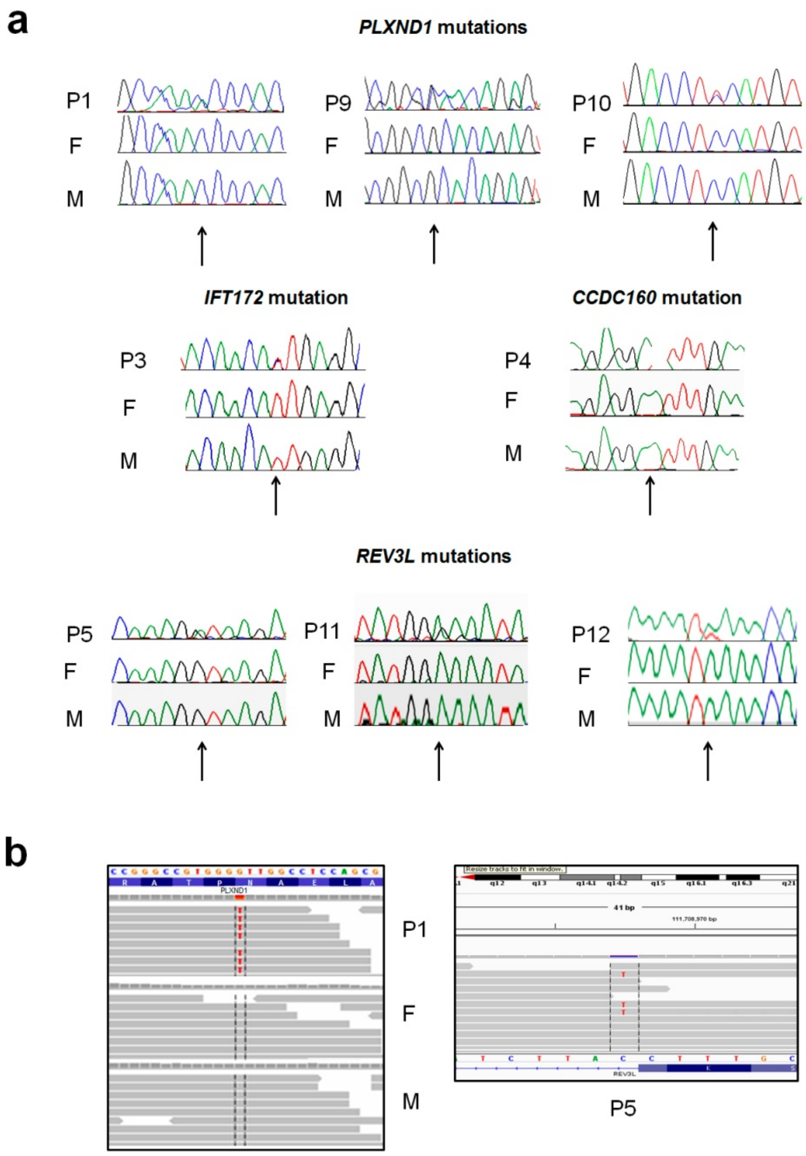

Supplementary Figure 1. (A) Sanger sequencing validation of the novo mutations in Möbius patients. The first row shows the *PLXND1* mutations in P1 (c.5685C>A), P9 (c.4454_4455GC>CA) and P10 (c.3018C>T). Patient 3 carries the c.1655T>C mutation in *IFT172*. Patient 4 carries a de novo c.501delA mutation in *CCDC160*. The last row shows the *REV3L* mutations in Patients P5 (c.1096+1G>A), P11 (c.1160A>G) and P12 (c.2662A>T). (B) Left. Reads from exome sequencing showing the presence of the *PLXND1* mutation in P1 and its absence in the parents. The mutation was present in 11 reads out of 31. Right. Reads from exome sequencing showing the presence of the *REV3L* mutation in patient P5.

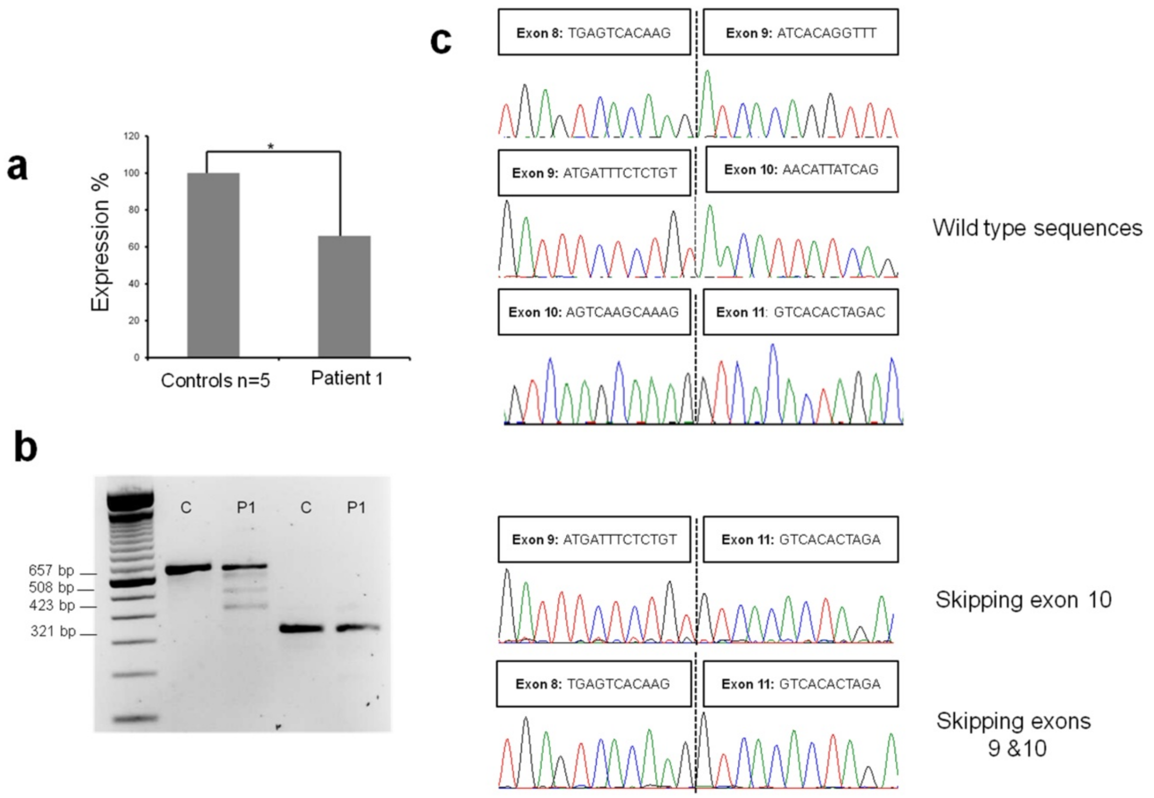

Supplementary Figure 2. *REV3L* Q-PCR and RT-PCR.

Effect of the c.1096+1G>A mutation on *REV3L* expression. (**a**) Shown are mRNA expression levels in EBV-LCLs cells in patient P5 compared to the average expression level of *REV3L* in five controls. (**b**) RT-PCR products of mRNA from patient P5 and one healthy control (C). with primers surrounding the canonical splice mutation (c.1096+1G>A) and control primers. Four bands of different sizes appear on the gel. The Sanger sequence of the first band corresponds to the correct size from the normal *REV3L* allele (657 bp). The sequence of the third band (508 bp) reveals skipping of exon 10 and the fourth band (423 bp) skipping of both exons, 9 and 10. Right two lanes: control RT-PCR (321 bp). The second band consists of heteroduplexes from the other products. (**c**) Sanger sequencing of RT-PCR products. The top three chromatograms are the cDNA sequences derived from the normal allele. The bottom two chromatograms are cDNA sequences of the 508 bp mutant product lacking exon 10 and of the 423 bp product lacking exons 9 and 10.

Homo sapiens	I L N E E A I S N L M E N S Q T F Q P L T Q I
Pan troglodytes	L I N E E A I L N L M E N S Q T F Q P L T Q L
Macaca mulatta	L I N E E A I L N L M E N S Q T F Q P L T Q L
Ratus norvegicus	L I N E E A I L N L I E N S Q T F Q P L T Q L
Mus musculus	L I N E E A I L N L I E N S Q T F Q P L T Q L
Canis lupus familiaris	L I N E E A I L N I V E N S Q S F H R L S Q L
Bos taurus	V V D E E A I L S M L E N S Q S F L Q L S Q V
Gallus gallus	T L S D Q T I L - - - - - - - - - - - - - - T
Xenopus laevis	I L N E E A I S N L M E N S Q T F Q P L T Q I
Tetraodon nigroviridis	L I N E E A I L N L M E N S Q T F Q P L T Q L

Homo sapiens	I M A A L E A N P T A R T Q L Q H K F E Q V
Pan troglodytes	I M A A L E A N P T A R T Q L Q H K F E Q V
Macaca mulatta	I M A A L E A N P T A R T Q L Q H K F E Q V
Ratus norvegicus	I M A A L E A N P T A R T Q L Q Y K F E Q V
Mus musculus	I M A A L E A N P T A R T Q L Q H K F E Q V
Canis lupus familiaris	I M T A L E A N P T A R T Q L Q H K F E Q V
Bos taurus	I M S A L E A N P T A R T Q L Q H K F E Q V
Ornithorhynchus anatinus	I V A A L E A N S T T R T Q L Q H K F E Q V
Gallus gallus	I V A A L D S N P T T K T Q L Q H K F E Q V
Xenopus laevis	I V T A L E T N P T T K G Q L Q H K F D Q V
Tetraodon nigroviridis	L D A T I S D D V W L Q A R F R R G P E D A

Homo sapiens	S A A K N P K L M L R R T E S V V E K M L T
Pan troglodytes	S A A K N P K L M L R R T E S V V E K M L T
Macaca mulatta	S A A K N P K L M L R R T E S V V E K M L T
Ratus norvegicus	S A A K N P K L M L R R T E S V V E K M L T
Mus musculus	S A A K N P K L M L R R T E S V V E K M L T
Canis lupus familiaris	S A A K N P K L M L R R T E S V V E K M L T
Bos taurus	S A A K N P K L M L R R T E S V V E K M L T
Gallus gallus	S A S K N P K L M L R R T E S V V E K M L T
Xenopus laevis	S A S K N P K L M L R R T E S V V E K M L T
Tetraodon nigroviridis	S A S K N P K L M L R R T E S V V E K M L T

Homo sapiens	T R I T I H G N D L H V G S E L Q V L V N D
Pan troglodytes	T R I T I H G N D L H V G S E L Q V L V L D
Macaca mulatta	T R I T I H G N D L H V G S E L Q V L V L D
Ratus norvegicus	T R I T I H G S D L N V G S M L Q V L V L D
Mus musculus	T R I T I H G S D L N V G S M L Q V L V L D
Canis lupus familiaris	T R I T I H G S D L H V G S E L Q V L V L D
Bos taurus	T R I T I H G S E L H V G S E L Q V L V L N
Gallus gallus	T R V T I R G S W L D V G S E L R V L V L S
Xenopus laevis	T R V T I S G T N L N V G R D V R V L F L K
Tetraodon nigroviridis	T T I T I T G E H L D I G S Q V R V M N T H

Supplementary Figure 3: Cross-species alignment of *REV3L* and *PLXND1* protein sequence surrounding the missense mutations. Cross species alignment of two regions of REV3L and PLXND1 showing the high amino-acid conservation of the missense variants of the p.(Glu387Gly) (P11, REV3L), p.(Asn1895Lys) (P1, PLXND1), p.(Arg1485Pro) (P9, PLXND1), and p.(Leu1006Leu) (P10, PLXND1) substitutions (in red) respectively. Residues with a grey background are conserved across the species.

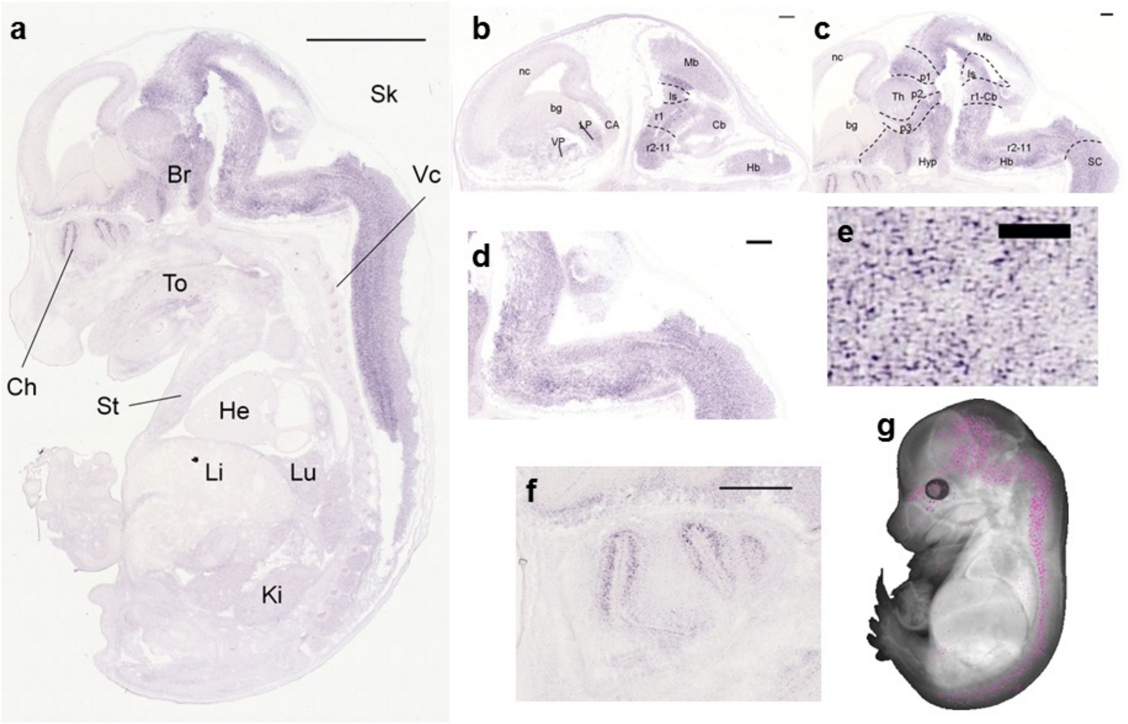

Supplementary Figure 4: *Rev3l* expression pattern in 14.5 d.p.c. embryonic mouse.

(**a-f**) Sagittal sections of 14.5 dpc mouse embryos processed respectively for ISH detection of *Rev3l* expression. The rostral is to the left. (**a**) Sagittal section of the entire mouse embryo to show specific *Rev3l* expression in brain (Br) and choanae (Ch). Other body structures are indicated such as the heart (He), liver (Li), lung (Lu), kidney (Ki), skull (Sk), sternum (St), tongue (To) and vertebral bodies (Vc) to show the absence of *Rev3l* expression. (**b-d**) Sagittal sections of embryonic mouse brain. The major antero-posterior subdivisions of the brain are outlined: diencephalon (prosomeres p1–p3), midbrain (M), isthmus (Is), cerebellum (rhombomeres r1–r11) and spinal cord (SC). *Rev3l* is expressed in ventral pallium (VP), lateral pallium (LP), hypothalamus (Hyp), p1-p3, midbrain, and it is broadly expressed in isthmus, rhombomeres and spinal cord. (**d-e**) Magnification of *Rev3l* expression centered at hindbrain positions. (**f**) Magnification of *Rev3l* expression centered at choanal position. In the sections processed for nonradioactive in situ hybridization, the positive cells appear with blue precipitate. (**g**) 3D reconstruction of *Rev3l* expression (in purple) in mouse at E14.5. These

figures correspond to images downloaded from the GenePaint server (http://www.genepaint.org). This server offers microphotographs of complete section series of mouse embryos processed for ISH with digoxigenin-labelled riboprobes. Other embryonic stages were not available in expression genes databases. Hb (hindbrain), nc (neocortex), Th (thalamus).

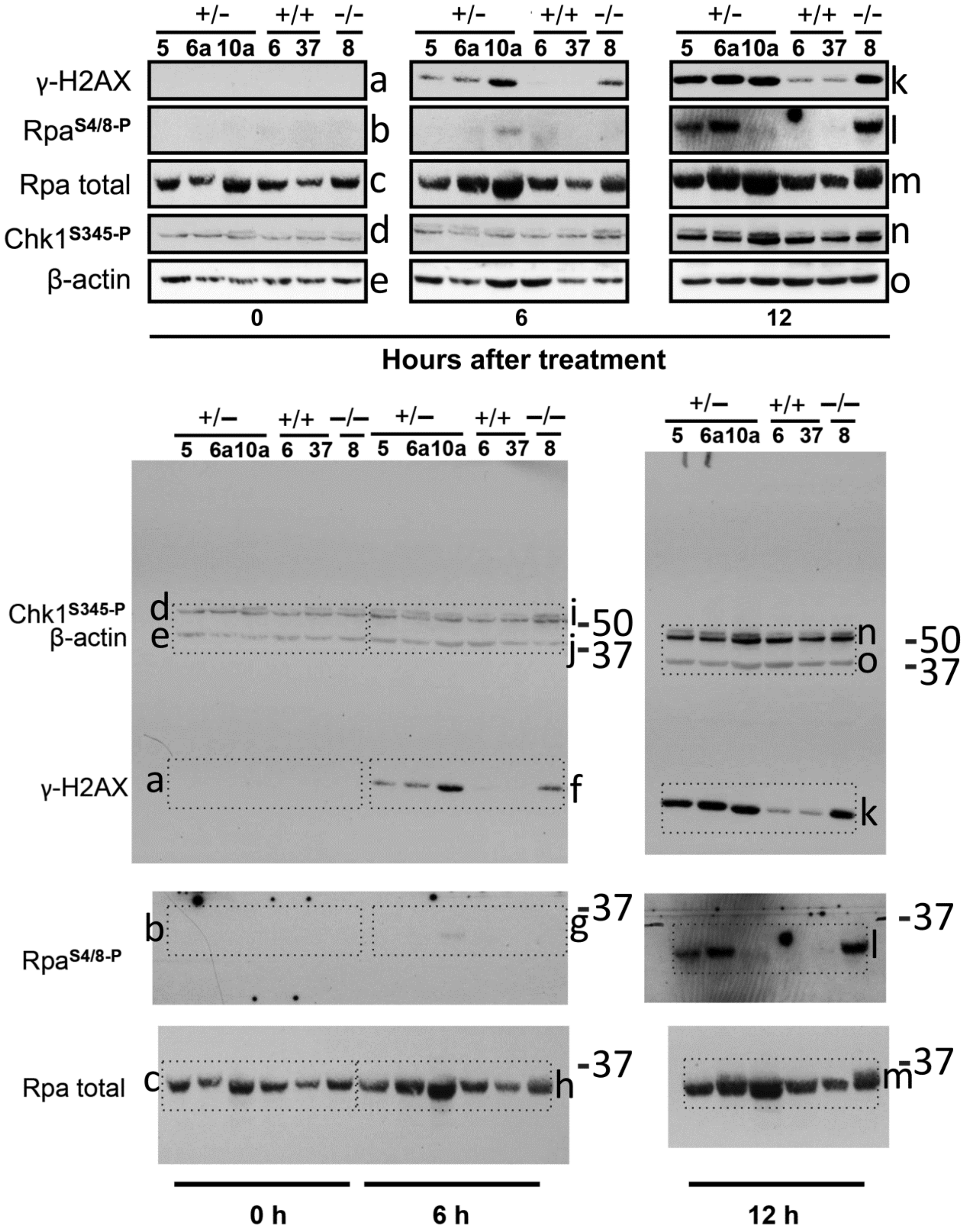

Supplementary Figure 5: Full blots of the panels shown in Figure 4 of the main text. The top panel shows a copy of Figure 4c in the main text. Each portion of the blot that was used for composing this figure has been lettered (a to m). These letters are also used to highlight the position on the original immunoblots from which they were derived (lower panel).

Supplementary tables

Supplementary Table 1. Filtering steps of exome sequencing data analysis.

	**Patient 1* (P1)**	**Patient 2 (P2)**	**Patient 3* (P3)**	**Patient 4* (P4)**	**Patient 5* (P5)**	**Patient 6 (P6)**	**Patient 7 (P7)**	**Patient 8 (P8)**
Total variants after QC	41468	39805	42288	41826	41522	40071	40604	41144
Exonic and canonical splice site variants	16736	16119	17068	16772	16244	15784	16011	16998
After exclusion of synonymous, known variants (dbSNP135) and variants present in in-house database	110	107	453	153	143	178	158	214
Possible stop and frameshift mutations	5	6	32	13	11	13	12	16
de novo candidates (or maternal X-linked)	2	-	1	1	1	-	-	-

QC Quality Control cut-off are ≥5 unique variant reads >5 and ≥15% variation reads.* Patients carrier of de novo mutations.

Supplementary Table 2. Relation of patient and mutations.

Patient	Case	Gene	Mutation	Technique
Patient 1	P1	*PLXND1; SPO11*	c.5685C>A; c.598T>C	NGS
Patient 2	P2	-		NGS
Patient 3	P3	*IFT172*	c.1655T>C	NGS
Patient 4	P4	*CCDC160*	c.501delA	NGS
Patient 5	P5	*REV3L*	c.1096+1G>A	NGS
Patient 6	P6	-		NGS
Patient 7	P7	-		NGS
Patient 8	P8	-		NGS
Patient 9	P9	*PLXND1*	c.4454_4455GC>CA	Sanger sequencing
Patient 10	P10	*PLXND1*	c.3018C>T	Sanger sequencing
Patient 11	P11	*REV3L*	c.1160A>G	Sanger sequencing
Patient 12	P12	*REV3L*	c.2662A>T	Sanger sequencing

NGS: Next Generation Sequencing .

Supplementary Table 3. De novo mutations in MBS patients

case	Gene	Chromo-some	Genomic position[1]	Mutation	Amino acid	Mutation type	PhyloP score	SIFT	Polyphen Prediction	CADD score
Patient 1	*PLXND1*	3	g.129,275,248	c.5685C>A	p.Asn1895Lys	Missense	0.771	Deleterious	Probably Damaging	Pathogenic 22.6
Patient 1	*SPO11*	20	g.55,910,931	c.712T>C	p.Cys238Arg	Missense	5.05	Tolerated	Probably Damaging	Pathogenic 16.7
Patient 3	*IFT172*	2	g. 27,693,832	c.1655T>C	p.Ile552Thr	Missense	4.706	Tolerated	Probably Damaging	Pathogenic 22.6
Patient 4	*CCDC160*	X	g.133,379,331	c.501del A	p.Glu167Aspfs*21	Frameshift	1.198	NA	NA	Non-Pathogenic 14.5
Patient 5	*REV3L*	6	g.111,708,967	c.1096+1G>A	See text	splice site	4.08	NA	NA	Pathogenic 23.8
Patient 9	*PLXND1*	3	g.129,284,263	c.4454_4455GC>CA	p.Arg1485Pro	Missense	3.84	Deleterious	Probably Damaging	Pathogenic 23.7
Patient 10	*PLXND1*	3	g.129,291,511	c.3018C>T	p.Leu1006Leu	Silent	-0.12	Tolerated	Neutral	Non-Pathogenic
Patient 11	*REV3L*	6	g.111,702,584	c.1160A>G	p.Glu387Gly	Missense	4.3	Not tolerated	Probably damaging	Pathogenic 27.9
Patient 12	*REV3L*	6	g.111,696,896	c.2662A>T	p.Lys888*	Nonsense	2.47	NA	NA	Pathogenic 35

[1] Genomic position is with respect to chromosome 2, 3, 6 and X in hg19.

Mutations are described numbered from the first nucleotide of the initiation codon in the nucleotide sequence NM_015103 (*PLXND1*), NM_012444 (*SPO11*), NM_001101357 (*CCDC160*), NM_015662 (*IFT172*) and NM_002912 (*REV3L*). SIFT (sorting intolerant from tolerant; http://sift.jcvi.org/). Polyphen is an amino acid substitution prediction method (http://genetics.bwh.harvard.edu/pph2/). Not tolerated, variants predicted to have a phenotypic effect. The CADD score (Combined Annotation Dependent Depletion) uses multiple annotations to predict pathogenecity[1].

Supplementary Table 4. Sequencing statistics of exome sequencing analysis

	Patient 1* (P1)	Patient 2	Patient 3*	Patient 4*	Patient 5*	Patient 6	Patient 7	Patient 8
Total reads	93,393,301	114,283,533	67,808,654	99,254,389	90,359,530	72,961,544	85,343,182	82,358,391
Total mapped bases	4,53Gb	5,49 Gb	3,24Gb	4,79Gb	4,33Gb	3,49Gb	4,08Gb	3,97Gb
Total mapped bases in targeted exons	3,706,404,046	3,672,657,533	2,515,750,529	3,884,801,966	3,319,497,862	2,625,060,286	3,131,034,093	3,183,131,055
Total mapped bases near targeted regions	6,8%	6,3%	8,6%	7,5%	9,2%	9%	9%	7,8%
Total mapped bases in regions	81,8%	66,8%	77,5%	81%	76,7%	75,1%	76,6%	80,2%
Total mapped bases outside regions:	11,3%	26,9%	14%	11,5%	14,2%	15,9%	14,4%	12%
% targets with at least 10x coverage	86,9	85,3%	79,9%	86,8%	82,2%	80%	81,2%	85,5%
Mean target coverage (fold)	72,1	72,4	48,5	73,6	64,1	50,6	60,6	59,9
Median target coverage (fold)	56,9	56,9	37,8	55,4	50,2	39,8	47,1	44,9

Patient 1 and 2 correspond to the patients in which we performed trio-exome sequencing. We performed exome sequencing in the isolated Patients 3-8. *Patients were we confirmed de novo mutations. Patient 1 is the carrier of the mutation in *PLXND1,* Patient 3 is the carrier of the mutation in *IFT172*, Patient 4 is the carrier of the mutation in *CCDC160* and patient 5 the carrier of the mutation in *REV3L*.

Supplementary Table 5. Clinical data of Möbius syndrome patients.

	P1	P5	P9	P10	P11	P12
**Gender**	F	M	F	M	F	F
**Cranial nerve defects**						
Right upper/lower facial palsy	++/-	++	-/-	++/++	++	+
Left upper/lower facial palsy	++/++	++	++/-	++/++	+	+
Abduction palsy RE/LE	++/++	++	++/++	++/++	++	
Horizontal gaze palsy	+		+	+	++	
Tongue malformations	+	+				
Lagophthalmos					+	+
Fibrosis of extra-ocular muscles			+			
**Oral dysfunction**						
Feeding problems	+		+	+		
Swallowing difficulties	+	+			+	
Palatal weakness	+	+				
Dysarthria	+	+		+		
Language delay	+	+				
**Craniofacial malformation**						
Microcephaly			+			
Epincanthic folds	+	+				
Flat nasal bridge	+	+	+			
Micrognatia	+			+		
External ear defects	+					
Dental defects	+		+	+	+	
Bifid uvula	+					
**Skeletal malformation**						
Clinodactyly	+					
Ectrodactyly		+				

Low set thumbs	+					
Rib deformities		+				
Hip defect	+					
Pes valgus	+					
Scoliosis	+					
Anisomelia	+	+				
**Poland anomaly**		+	+			
**Motor retardation**	+	+		+		+
Clumsy motor performance	+	+				
Dysdiadochokinesis	+		+			
Hypotonia		+				
**Sensory disturbances**						
Hearing loss		+				
Elevated pain threshold	+					
**Autism**	+					
**Vascular defects**						
Cardiac defect		+				
Facial Hemangiomas	+					

The following abbreviations are used: F, Female; RE, right eye; LE, left eye; ++, paralysed; + paretic or affected.

Supplementary Table 6. Primers sequences used in this study.

Sanger sequencing Exon	Foward Primer	Reverse primer
PLXND1-Exon1a	ggcggaaaagagaaaacag	gctgatagaggcggttgac
PLXND1-Exon1b	ggtcgaaggtgaagagcttg	ggtcgaaggtgaagagcttg
PLXND1-Exon1c	tccatctaccagggcttctg	ggtcgaaggtgaagagcttg
PLXND1-Exon 2	tcatctccttggaacagctc	agtggcaaaaacagacttgc
PLXND1-Exon 3	tcctgaagggtccaagacag	gcagaggaacagcgtgtgta
PLXND1-Exon 4&5	agtgttgaccctcagtttcc	atttgtcttcaggtccttgg
PLXND1-Exon 6&7	gggtcacatgtagggcatag	tgtgctggtgaatgaacaac
PLXND1-Exon 8	ctcttgttagcaggcagctc	gcaaaggcattgaaacattc
PLXND1-Exon 9	caggactgaaccaggcag	gaaagaaaagcaaagcccac
PLXND1-Exon 10&11	aaaggcctgacagttcgac	ggcaaccactggaatgag
PLXND1-Exon 12	gaaggggctaagaacctcag	ggtggctttagtgatcatgg
PLXND1-Exon 13	tcctctaagcagtgggtctg	tgattttcctcagagcaagg
PLXND1-Exon 14&15	gaactgtgggaaactgatgg	cttggctcaaggatgtcac
PLXND1-Exon 16&17	actgggcagaagatgagttg	aggaagaggggacagagatg
PLXND1-Exon 18	atctctgtcccctcttcctc	ctgtcctgctccttctgtg
PLXND1-Exon 19	agcctctcaccctcgttatc	cagtgaattcaggtgctgg
PLXND1-Exon 20	ctgggtgcctaacctgttc	ctgggtgcctaacctgttc
PLXND1-Exon 21&22	cagatggacaacctggagtc	tctcgaggttcttctcgatg
PLXND1-Exon 23	catgacagggatcaatgc	catgacagggatcaatgc
PLXND1-Exon 24	taatgagctccccatccc	caggaagggctggaatatg
PLXND1-Exon 25	ggttgctgtggacgtagaag	ctgcataacaggtcctacgg
PLXND1-Exon 26&27	cctgacacggggtctctg	ggcagcaactcactgacctt
PLXND1-Exon 28&29	tacatggagagagcgagagg	caggaaatcagcttgaggag
PLXND1-Exon 30&31	caccctcatcctgtccaag	acgctctggaagaagttgtc
PLXND1-Exon 32	atgtggcttcctcattcagc	agctgggaaatggcagag
PLXND1-Exon 33	tctctcctccacaactgtcc	ggtgtctccctgtctgaatg
PLXND1-Exon 34	gtcacttatgtggcaggagg	tccctgcccttactccag
PLXND1-Exon 35	cgctgaactgttctgtggg	agtgacatccgtccattagg
PLXND1-Exon 36	actcgtggtagacgttcagg	ttcccagtctgagtcacagg

REV3L-Exon1	gagaaagccccttctcgg	gacaaacctgtagtgcctcc
REV3L-Exon 2	actggttactttgaactatggg	ggttgggatcagaggtttg
REV3L-Exon 3	taagactgaatttggccagc	cagtttgttcttgaacttagagatg
REV3L-Exon 4	ttccggtaaaattgagatgg	tgctgagctgtgactttgtc
REV3L-Exon 5	aggtcagatcgagaccatcc	ggggagatgttttcttaatttg
REV3L-Exon 6	caggttttctgggtacaagc	ggggagatgttttcttaatttg
REV3L-Exon 7	gggataatttgtccactttattacac	aggagggagggagagacttc
REV3L-Exon 8 & 9	tgtgtgattctatggtaaaactacag	aattatgattcagttgtactgcac
REV3L-Exon 10	atggtaatgcttccaaatttac	ttagcatactttgcttaaaggc
REV3L-Exon 11	cactgcactccaactccag	cttattctgaaaagcagattatcc
REV3L-Exon 12	agaatgcaggaattgtggtg	ttttggtaatgaggcaaataaac
REV3L-Exon 13[a]	tcttggtgtgggaaaatgtc	ttcaattcctgacagttggc
REV3L-Exon 13[b]	tgggcaaaaattctttcaac	ttttctcgtttcatttctgg
REV3L-Exon 13[c]	gtcactttggagatggaacg	gcattagacaagagtcatagcc
REV3L-Exon 13[d]	aatgctgaaaccgaagattg	ttatatcatgaatttgttgggc
REV3L-Exon 13[e]	ggctctgctgtagatcatcc	caggttacccagtagttacataattg
REV3L-Exon 13[f]	tgcattgcaaagacagtcag	tgagaaagatggcttccataac
REV3L-Exon 13[g]	gtaacgtccccaagaaaacc	agctttgctgtgaagctgac
REV3L-Exon 13[h]	tgtttcttagcctcccacag	tgtctgccagtaatctgtgg
REV3L-Exon 14[a]	acctgcacaatatagagggg	gacaaacctgtagtgcctcc
REV3L-Exon 14[b]	gacaaacctgtagtgcctcc	agtttgactacatcccgtgg
REV3L-Exon 15	ggttgggatcagaggtttg	acagcagaaagacagttgc
REV3L-Exon 16	cagtttgttcttgaacttagagatg	ttgtctcaaagatatgcaaataatg
REV3L-Exon 17	tgctgagctgtgactttgtc	gaattcaatatactcctcccttg
REV3L-Exon 18	ggggagatgttttcttaatttg	tctcctacccctacctcagc
REV3L-Exon 19	ggggagatgttttcttaatttg	ttcgctttcttcctacctttac
REV3L-Exon 20	aggagggagggagagacttc	ttcgctttcttcctacctttac
REV3L-Exon 21	aattatgattcagttgtactgcac	gttttgccatgttgctcag
REV3L-Exon 22	ttagcatactttgcttaaaggc	caactggcctgaacaatacac
REV3L-Exon 23	cttattctgaaaagcagattatcc	gccggaaagttggcatag
REV3L-Exon 24	ttttggtaatgaggcaaataaac	atagggaaggtgtccagagc

REV3L-Exon 25	ttcaattcctgacagttggc	caaggtgactggtttgttc
REV3L-Exon 26	ttttctcgtttcatttctgg	aggattcaactctgggctg
REV3L-Exon 27	gcattagacaagagtcatagcc	cgtaaggactggaaaggactc
REV3L-Exon 28	ttatatcatgaatttgttgggc	agacatgagccagtgaccc
REV3L-Exon 29	caggttacccagtagttacataattg	acctagatgcatgtttaaggg
REV3L-Exon 30	tgagaaagatggcttccataac	tcagtgaggcttcatgtgc
REV3L-Exon 31	agctttgctgtgaagctgac	tgaaagcaggagtgcctatc
REV3L-Exon 32	tgtctgccagtaatctgtgg	atggccattcttgttgtagc
CCDC160-Exon2[a]	tagattcttcacccgtgagc	gcaggtagagttatcttctgtgc
CCDC160-Exon2[b]	catttcaaagaatgaaacagacac	ttgtggatgacttctttggc
CCDC160-Exon2[c]	ttttcaagaaacagagacgg	aaagagaaagcagaagcttgg
**Q-PCR**		
GUSB	agagtggtgctgaggattgg	ccctcatgctctagcgtgtc
PPIB	cggaaagactgttccaaaaac	gattacacgatggaatttgctg
REV3L	tcgtctggacattgaagctc	gactcaggttggctcatttg
***REV3L* RT-PCR**		
REV3L-Exon 4-10	gctgtcaagttccgaaaagc	ttgggtcaaaggctgaaaag
REV3L-Exon 13	acagaaaagccaagcctcag	tgcgtttggctctaagtgtg

References

1. Kircher, M. *et al.* A general framework for estimating the relative pathogenicity of human genetic variants. *Nat. Genet.* **46**, 310-315 (2014).
